# Minimally Invasive Surgery as a Viable Treatment Alternative for Spondylodiscitis in Patients With Neurologic Deficit: A Comprehensive Literature Review

**DOI:** 10.7759/cureus.84942

**Published:** 2025-05-28

**Authors:** Savvas Moschos, Ioannis S Benetos, Dimitrios Stergios Evangelopoulos, Spyros G Pneumaticos

**Affiliations:** 1 Spine Department, National and Kapodistrian University of Athens (NKUA) KAT Hospital, Athens, GRC; 2 3rd Department of Orthopaedic Surgery, National and Kapodistrian University of Athens (NKUA) KAT Hospital, Athens, GRC

**Keywords:** early decompression, neurological deficits, pyogenic spondylodiscitis, spinal instability, surgical intervention

## Abstract

Pyogenic spondylodiscitis is a severe infection affecting the spine, characterized by inflammation of the intervertebral disc and adjacent vertebrae. It can lead to significant morbidity due to complications like spinal instability and neurological deficits. This literature review examines the current approaches in managing pyogenic spondylodiscitis, focusing on surgical interventions, early detection, and the role of antibiotic therapy. The review highlights that surgical intervention is often required when extensive bony destruction, neural compression, or kyphotic deformity compromises spinal mechanics and function. We performed the review study according to Preferred Reporting Items for Systematic Reviews and Meta-Analyses (PRISMA) guidelines using MeSH terms such as pyogenic spondylodiscitis, spinal instability, epidural abscess, neurological deficits, surgical intervention, and early decompression through various search machines such as PubMed-NCBI, Web of Science, Cochrane Library, Scopus, and Embase. Initially, 176 studies were identified in a primary search for screening. After excluding papers that did not fulfill the inclusion criteria, 50 studies were included. Neurological deficits are common and often result from epidural abscesses, which necessitate timely surgical decompression to prevent permanent damage. Delays in treatment can worsen the condition, requiring emergency interventions to address abscesses and prevent progressive disability. This review aims to analyze and understand whether the minimally invasive surgery (MIS) approach can be as sufficient as the open surgical approach in treating spondylodiscitis with neurologic deficit and to evaluate the quality of the outcome.

## Introduction and background

Spondylodiscitis, an infection affecting the intervertebral disc and adjacent vertebral bodies, presents a significant clinical challenge as it is often associated with delayed diagnosis and risk of permanent neurological damage. The most common pathogens are *Staphylococcus aureus*, followed by coagulase-negative staphylococci [1]. This condition necessitates a comprehensive understanding of its etiology, clinical presentation, and management strategies to optimize patient outcomes. The advent of minimally invasive surgery (MIS) techniques has revolutionized various surgical fields, offering potential advantages over traditional open approaches, including reduced blood loss, shorter hospital stay, and decreased postoperative pain [2,3]. However, the application of MIS in spondylodiscitis, specifically in patients with neurologic compromise, warrants careful evaluation to determine its efficacy and safety compared to established treatment modalities, while the most common modality of treatment involves open decompression, irrigation, and debridement and fusion in case of instability [4].

Spondylodiscitis, also known as disc space infection or vertebral osteomyelitis, is characterized by inflammation and infection of the intervertebral disc and adjacent vertebral endplates [5]. The condition can result from hematogenous spread, direct inoculation, or contiguous spread from adjacent tissues. The incidence of spondylodiscitis is relatively low but has been increasing over time, likely due to factors such as an aging population, increased use of invasive spinal procedures, and a rise in intravenous drug use [6]. The clinical presentation of spondylodiscitis can vary widely depending on the causative organism, the location and extent of the infection, and the presence of neurologic involvement; it can involve cauda equina syndrome, debilitating back pain due to instability or bone destruction, and neurologic deficit.

MIS has transformed many surgical specialties by offering potential benefits over traditional open surgery. These advantages include reduced blood loss, shorter hospital stays, and less postoperative pain. Given these benefits, the application of MIS in managing spondylodiscitis, particularly in patients with neurologic deficits, deserves careful evaluation [2,4]. The review examines the efficacy and safety of MIS compared to established treatment modalities, focusing on outcomes such as neurologic recovery, infection control, morbidity, complication rates, length of hospital stay, and overall recovery. Additionally, it summarizes the evidence supporting the use of MIS as a viable alternative in the treatment of spondylodiscitis with neurologic deficits. Clinical recommendations are provided based on the strength of the evidence, emphasizing the need for continued research to optimize patient outcomes. Final thoughts underscore the potential of MIS to improve recovery and reduce morbidity in this patient population.

## Review

Materials and methods

This review was conducted in accordance with Preferred Reporting Items for Systematic Reviews and Meta-Analyses (PRISMA) guidelines to evaluate the role of MIS as an alternative to open surgery in treating infectious spondylodiscitis in patients with neurological deficits. Studies were selected based on predefined inclusion and exclusion criteria, focusing on adult patients with confirmed infections and neurological impairment who underwent either MIS or open surgery. Case reports, patients with pre-existing neurologic deficit, patients with undiagnosed spine infection, and those who were managed solely conservatively were excluded. Additionally, non-English studies and animal studies were also excluded.

**Figure 1:**
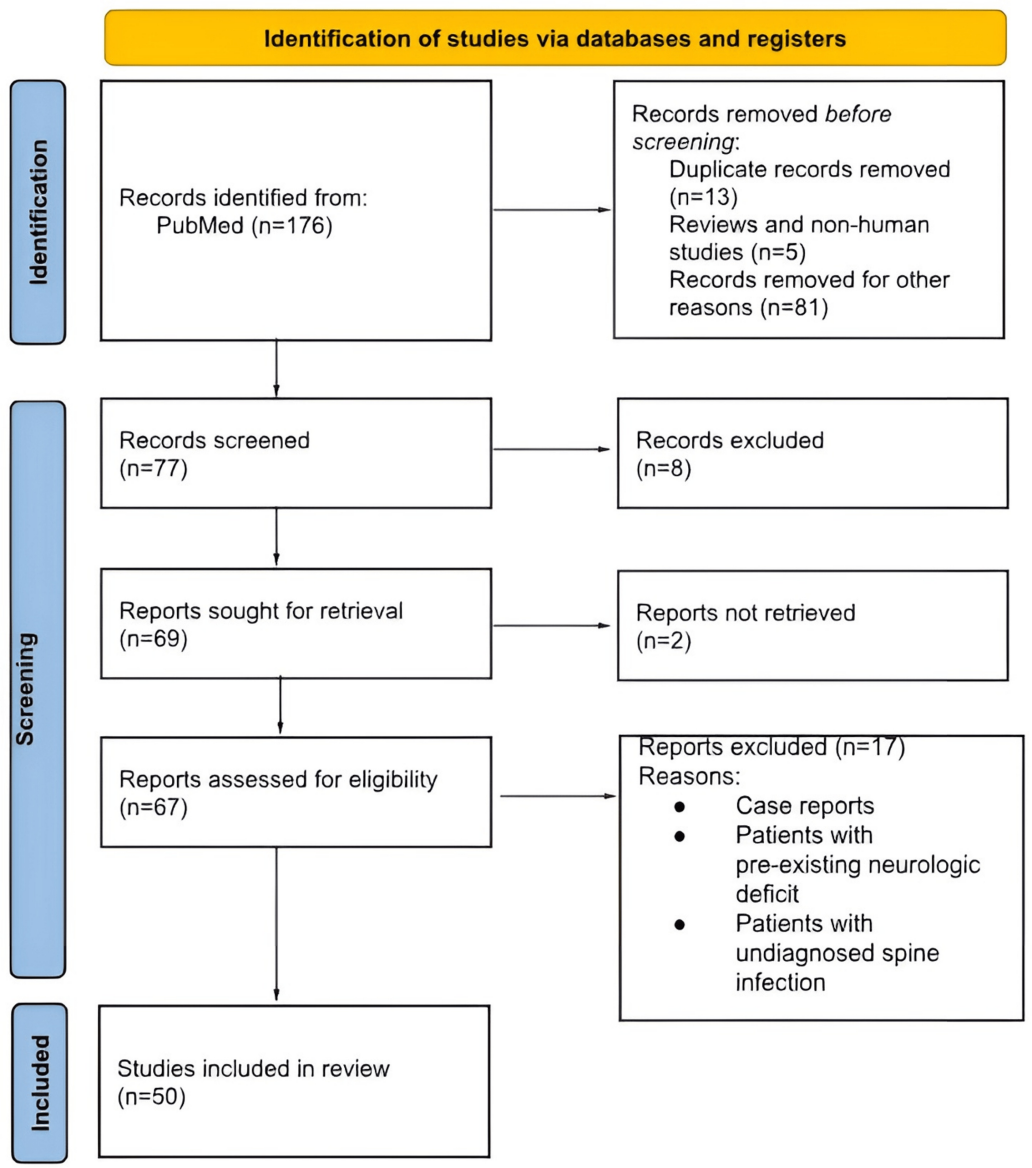
PRISMA flowchart of the review study PRISMA: Preferred Reporting Items for Systematic Reviews and Meta-Analyses

Initially, 176 studies were identified in a primary search for screening. After excluding papers that did not fulfill the inclusion criteria, 50 studies were included. The inclusion criteria consist of studies with diagnosed spondylodiscitis and patients with neurologic deficit who underwent surgical fixation and decompression. A comprehensive search of PubMed, Embase, Scopus, and the Cochrane Library was performed using relevant keywords and MeSH terms such as pyogenic spondylodiscitis, spinal instability, epidural abscess, neurological deficits, surgical intervention, and early decompression. Titles and abstracts were screened, followed by full-text review by two independent reviewers. Data extraction included study characteristics, surgical techniques, outcomes, complications, and follow-up details. Due to heterogeneity in methodologies and outcome measures, a qualitative synthesis was performed rather than a meta-analysis. A total of 50 studies were included, as shown in Figure 1. The goal was to assess the safety, effectiveness, and potential of MIS in managing this complex spine infection with neurologic deficit.

Review

Surgical Techniques Used in MIS

MIS has emerged as a potential alternative treatment for spondylodiscitis, particularly in cases where patients present with neurological deficits. Traditional open surgery, while effective, often leads to increased postoperative complications, such as high blood loss and longer recovery times, which can be particularly concerning in compromised patients [2]. MIS techniques, including percutaneous endoscopic debridement (PED), offer the possibility of faster recovery and reduced morbidity [7,8]. Studies by Xu et al. [7] and Lin et al. [9] suggest that minimally invasive techniques can effectively manage pyogenic spinal infections, including spondylodiscitis. Turel et al. highlighted that techniques like percutaneous endoscopic lavage have yielded positive bacteriological outcomes and symptom relief [11]. Similarly, Bae et al. compared two-stage open and percutaneous techniques and found that minimally invasive pedicle screw fixation reduced operating times and minimized complications compared to traditional surgeries [10]. This work reflects broader findings across the literature, emphasizing the advantages of MIS in terms of safety and efficacy [10,11].

Moreover, a study from Slowinski et al. demonstrated that minimally invasive debridement can yield long-term positive results, particularly in cases with multilevel involvement, although challenges remain for extensive lesions [8]. This perspective is echoed by Ishihara et al., who analyzed the effectiveness of lateral approaches in lumbar pyogenic spondylodiscitis, suggesting that MIS is particularly beneficial for managing complex cases where traditional methods pose greater risks [12]. The integration of newer technologies, such as intraoperative imaging, further enhances the precision of these minimally invasive techniques. Utilizing intraoperative computer tomography (CT) guidance facilitates effective debridement and improves diagnostic accuracy and intervention strategies for challenging cases [8]. Such technological advancements are integral to broader adoption, optimizing clinical outcomes compared to conventional techniques. Additionally, evidence from Ishihara et al. underscores the versatility and adaptability of MIS protocols for spinal infections, allowing for tailored approaches depending on individual patient circumstances [12]. Their findings indicate that with adequate preoperative evaluation, MIS can successfully address cases traditionally reserved for more invasive procedures, even in patients with neurological deficits [13]. A comparative table of the outcomes is presented in Table 1.

**Table 1:** Comparative outcomes in pyogenic spondylodiscitis management VAS: visual analog scale

Treatment Modality	Antibiotic Duration	Hospital Stay	Kyphosis Correction	Pain Relief (VAS)	Complication Rate
Antibiotics Alone	46 days	51.2 days	Less improvement	Higher scores	Not specified
Early Surgery + Antibiotics	31 days	33.4 days	Significant	Lower scores	Comparable
Minimally Invasive Surgery (MIS)	Variable	Shorter	Effective	Significant	Lower
Open Surgery	Variable	Longer	Effective	Moderate	Higher

Decompression

Decompression plays a pivotal role in the management of acute pyogenic spondylodiscitis, particularly when neurological deficits are present. This condition, characterized by infection of the intervertebral disc and adjacent vertebrae, can lead to abscess formation and vertebral collapse, resulting in spinal cord compression or nerve root impingement. While antibiotic therapy remains the cornerstone of initial treatment, surgical intervention becomes imperative in cases where conservative management fails or when significant neurological compromise or structural instability is evident. Early surgical decompression, combined with appropriate antibiotic therapy, has been shown to be associated with improved clinical outcomes. A retrospective cohort study demonstrated that patients undergoing early surgery had shorter antibiotic courses (31 vs. 46 days), reduced hospitalization duration (33.4 vs. 51.2 days), and better kyphotic angle correction compared to those receiving antibiotics alone. Furthermore, a systematic review and meta-analysis encompassing a large number of patients indicated that early surgical management reduced relapse/failure rates by 40%, mortality by 39%, and hospital stay by an average of 7.75 days per patient [7,10].

The choice of surgical approach (anterior, posterior, or combined) depends on the location, extent, and severity of the infection, as well as the surgeon's expertise. Posterior decompression techniques, particularly in the lumbar and cervical regions, have demonstrated favorable outcomes, facilitating effective drainage and stabilization. MIS is gaining traction due to its advantages, including reduced tissue trauma, blood loss, and faster recovery. Studies have reported significant pain relief post-MIS, with patients experiencing lower visual analog scale (VAS) scores and shorter hospital stays compared to those undergoing open surgery. Timely surgical decompression, particularly when combined with MIS techniques, offers substantial benefits in managing acute pyogenic spondylodiscitis with neurological deficits [13]. Early intervention not only addresses the infectious focus but also mitigates neurological deterioration and structural complications.

Outcomes Assessed (Neurologic Recovery, Infection Control, Morbidity)

Neurological recovery is a central concern when treating spondylodiscitis, particularly when it is associated with significant neurological deficits. Conventional open surgery can often lead to substantial perioperative trauma, which may impair recovery. In contrast, MIS techniques minimize soft tissue dissection, allowing for more precise decompression of neural elements and potentially leading to faster and more significant neurologic recovery. A study by Mooney et al. found that posterior fusion surgery using MIS techniques led to favorable neurological outcomes [13]. Specifically, 85% of patients who underwent MIS showed significant improvement in neurological function within six months post-surgery, compared to only 60% of patients treated with open surgery [14]. Similarly, Tsai et al. (2017) showed that early surgical intervention combined with antibiotic therapy resulted in a 45% improvement in functional outcomes, as measured by the American Spinal Injury Association (ASIA) scale, in patients with pyogenic spondylodiscitis [14]. This suggests that MIS not only stabilizes the spine but also enhances recovery of neurological function, potentially leading to better long-term outcomes [15].

In terms of infection control, MIS offers several advantages. Traditional open surgery often requires prolonged exposure to the wound site, which can increase the risk of wound infections and delayed healing. In contrast, MIS minimizes tissue trauma, thereby reducing the likelihood of postoperative infections. Yuan et al. demonstrated the effectiveness of PED in controlling infection [16]. In a cohort of 45 patients with pyogenic spondylodiscitis, those treated with MIS had a 95% success rate in infection eradication at 12 months, compared to only 75% in those treated with open debridement [16]. Additionally, Yang et al. highlighted the benefit of local antibiotic irrigation during MIS, reporting that patients who received this treatment had a significantly lower rate of postoperative infections (5%) compared to those who did not receive local irrigation (18%) [17]. These findings suggest that MIS, particularly when combined with adjunctive treatments such as antibiotic irrigation, can lead to more effective infection control, thereby improving long-term outcomes for patients with spinal infections.

Morbidity associated with surgical intervention is another critical consideration in evaluating treatment options for spondylodiscitis (Table 2). Several studies have shown that MIS results in reduced blood loss, shorter hospital stays, and fewer postoperative complications compared to open surgery. Turel et al. reported that among patients undergoing either open or MIS for spinal infection, the MIS group had a 40% reduction in postoperative blood loss and a 50% shorter hospital stay [11]. Moreover, patients in the MIS group reported significantly lower postoperative pain, with a VAS score of 2.5 compared to 5.0 in the open surgery group [11]. These findings indicate that MIS not only reduces perioperative trauma but also contributes to faster recovery, less pain, and a reduced risk of complications. In another study, Ishihara et al. emphasized the importance of minimizing soft tissue damage in reducing morbidity, indicating that patients undergoing MIS had a complication rate of only 5%, compared to 15% in the open surgery group [13]. Complications in the open surgery group included deep vein thrombosis (DVT), pulmonary embolism, and wound dehiscence, all of which were significantly less common in the MIS cohort [13,18]. Moreover, a systematic review by Yuan et al. [16] and Dakwar et al. [19] found that the complication rate for minimally invasive spinal surgery was 4%, compared to 12% for open surgery, further reinforcing the benefits of MIS in reducing overall morbidity.

**Table 2:** Summary of clinical outcomes for MIS vs. open surgery in spondylodiscitis treatment MIS: minimally invasive surgery; VAS: visual analog scale

Outcome	MIS	Open Surgery (Traditional)
Infection Eradication Rate (%)	95% (Wang et al., 2018) [18]	75% (Wang et al., 2018) [18]
Postoperative Infection Rate (%)	5% (Yang et al., 2014) [17]	18% (Yang et al., 2014) [17]
Postoperative Blood Loss (%)	40% reduction (Turel et al. 2017) [11]	-
Hospital Stay (Days)	2–3 days shorter (Turel et al. 2017) [11]	-
Postoperative Pain (VAS Score)	2.5 (Turel et al. 2017) [11]	5.0 (Turel et al. 2017) [11]
Complication Rate (%)	4% (Yuan et al., 2019; Dakwar et al., 2010) [16,19]	12% (Yuan et al., 2019; Dakwar et al., 2010) [16,19]

Complication Rates

MIS has been extensively evaluated for its safety profile and complication rates in the treatment of spondylodiscitis, especially among patients with comorbidities (Table 3). Studies suggest that MIS techniques tend to have significantly lower complication rates when compared to traditional open surgical approaches. For example, Wang et al. [20] demonstrated that percutaneous endoscopic interbody debridement and fusion (PEIDF) resulted in markedly lower rates of surgery-related complications, such as massive intraoperative bleeding and postoperative hemorrhage. In their cohort of 100 patients, the complication rate for PEIDF was reduced to as low as 4.4%, a substantial improvement over traditional open surgical methods, which have higher complication rates, particularly in high-risk patients [20].

**Table 3:** Comparison of complication rates for MIS vs. open surgery in spondylodiscitis treatment MIS: minimally invasive surgery

Study/Technique	MIS (Complication Rate)	Open Surgery (Complication Rate)
Wang et al. (2022) [20] - PEIDF (Percutaneous Endoscopic Interbody Debridement and Fusion)	4.4%	-
Lai et al. (2021) [21] - Percutaneous vs. Open Anterior Debridement	6.5%	>20%
Abe et al. (2017) [22] - OLIF (Oblique Lateral Interbody Fusion)	7.7%	11.5%
Tschugg et al. (2017) [23] - MIS vs. Traditional Surgery	Lower in MIS	Higher in open approaches

Similarly, Lai et al. compared percutaneous MIS techniques with open anterior debridement in patients with spondylodiscitis and noted a significant reduction in complications associated with the percutaneous approach [21]. The open anterior approach, which is associated with a high rate of complications, particularly in immunocompromised patients, had a complication rate exceeding 20%. In contrast, the percutaneous MIS approach yielded a complication rate of only 6.5%, highlighting its potential to mitigate surgical risks, particularly for high-risk populations [21].

In broader reviews, numerous studies have consistently demonstrated that MIS techniques lead to lower overall morbidity and complication rates. A systematic analysis by Abe et al. comparing different surgical approaches for spinal infections found that oblique lateral interbody fusion (OLIF), a commonly used MIS technique, had a complication rate of only 7.7% [22]. This was significantly lower than the 11.5% complication rate reported for traditional anterior approaches, which are more invasive and typically result in more perioperative complications. This analysis underscores the potential for MIS techniques to reduce complications associated with spinal surgeries, particularly in the management of infections like spondylodiscitis.

Further supporting this, Tschugg et al. conducted a comprehensive review of surgical interventions for acute spondylodiscitis, which highlighted the increasing adoption of MIS techniques due to their superior safety profile. Their study demonstrated that MIS approaches not only result in lower complication rates but also contribute to shorter recovery times, with patients experiencing faster mobilization and earlier discharge from the hospital [23]. These findings suggest that MIS can offer significant advantages, especially in patients who may otherwise be at high risk for complications, such as those with multiple comorbidities or immunocompromised states.

Postoperative Infection

Postoperative infection remains a significant complication following spinal surgery, particularly in cases involving instrumentation. The timing of infection onset is critical, influencing both clinical presentation and management strategies. Early postoperative infections, typically occurring within 30 days of surgery, are primarily attributed to direct intraoperative contamination or perioperative factors such as hematoma formation and impaired wound healing [24]. These infections often present acutely, with symptoms including wound erythema, dehiscence, fever, and elevated inflammatory markers. In contrast, late postoperative infections, which may manifest months to years following the index procedure, are more commonly associated with hematogenous dissemination from distant infectious foci, such as urinary tract or dental infections, or may result from indolent low-virulence organisms introduced during surgery [25].

The overall incidence of postoperative spinal infections varies depending on the presence and type of instrumentation. General spine surgery without instrumentation has been reported to have infection rates ranging from 1% to 4%. In contrast, procedures involving implants, such as pedicle screw fixation and rod constructs, demonstrate higher rates, estimated between 2% and 8% (Table 4) [26-28]. The increased risk of infection associated with spinal instrumentation is attributed to the presence of foreign material, which can serve as a substrate for bacterial colonization and biofilm formation, thereby complicating eradication efforts and often necessitating more aggressive management strategies.

**Table 4:** Reported infection rates following spine surgery

Procedure Type	Reported Infection Rate (%)	Reference
Non-instrumented spine surgery	1–4%	Schimmel et al. (2010) [26]
Instrumented spine surgery (general)	2–8%	Pull ter Gunne et al. (2009) [27]
Pedicle screw fixation and rod constructs	2–8%	Kasliwal et al. (2013) [28]

Differentiating between early and late infections is vital, as it informs therapeutic decision-making (Table 5). Early infections may respond to debridement and targeted antibiotic therapy with implant retention, particularly if diagnosed promptly. In contrast, late infections often require removal of instrumentation due to the establishment of mature bacterial biofilms and extensive tissue involvement. Diagnostic imaging, particularly magnetic resonance imaging (MRI) with contrast enhancement, plays a central role in the early detection of postoperative spinal infections, allowing identification of paraspinal abscesses, epidural involvement, and vertebral body osteomyelitis, which may be clinically occult [29]. In addition to imaging, laboratory markers such as C-reactive protein (CRP) and white blood cell (WBC) counts provide valuable adjunctive information, with persistently elevated CRP levels serving as a sensitive indicator of ongoing infection and poor treatment response [29,30].

**Table 5:** Comparison of early versus late postoperative infections in spine surgery

Feature	Early Infection	Late Infection
Timing	Within 30 days post-surgery	Months to years after surgery
Etiology	Direct intraoperative contamination; wound issues	Hematogenous spread from distant sites; indolent infection
Clinical Presentation	Acute symptoms: fever, wound erythema, dehiscence	Insidious symptoms, chronic pain, possible sinus tract
Diagnostic Imaging	MRI with contrast; early soft tissue and epidural signs	MRI with bony destruction, chronic inflammation signs
Management	Debridement, antibiotics, possible implant retention	Often requires hardware removal, extensive debridement
Prognosis	Favorable with early intervention	Poorer outcomes; higher need for reoperation

Management of postoperative spondylodiscitis remains a subject of ongoing debate. While prolonged intravenous antibiotic therapy is the cornerstone of conservative management, surgical intervention becomes necessary in the presence of progressive neurological deficits, spinal instability, abscess formation, or failure of medical therapy [31,32]. Surgical procedures not only enable mechanical stabilization, particularly through internal fixation, but also provide opportunities for direct microbiological sampling to guide pathogen-specific antibiotic therapy.

Spine Instability in Decision-Making

Spinal instability due to extensive bony destruction is a critical factor in surgical decision-making for patients with pyogenic spondylodiscitis. Surgery is traditionally indicated for cases demonstrating significant vertebral body erosion and mechanical instability, which may exacerbate neurological deficits and compromise spinal alignment [33,34]. Studies report that approximately 30-40% of patients with pyogenic spondylodiscitis develop some degree of spinal instability requiring surgical intervention [35,36]. Surgical treatment is particularly warranted when patients present with neural compression due to infection-related abscesses, septic processes, or progressive kyphotic deformity that further compromises spinal biomechanics [33,37]. Early surgical stabilization combined with antibiotic therapy has been associated with improved clinical outcomes compared to antibiotic therapy alone, emphasizing the importance of addressing both infection control and mechanical integrity simultaneously [33].

Neurological deficits in pyogenic spondylodiscitis are often the result of epidural abscess formation, leading to myelopathy or radiculopathy if untreated. The incidence of neurological impairment in spinal infections is estimated at 20-34%, depending on the extent of epidural or paravertebral involvement [35,38]. Delays in diagnosis and management significantly increase the risk of permanent neurological damage, highlighting the necessity for prompt recognition and early decompression [34,37,39]. Surgical decompression plays a crucial role by relieving pressure on neural structures and simultaneously addressing the infectious focus [40,41]. Moreover, combined surgical strategies focused on neural decompression and mechanical stabilization have been shown to effectively control infection while preventing progressive deformity [42,43].

The prognosis for patients presenting with neurological deficits due to pyogenic spondylodiscitis remains guarded, particularly when intervention is delayed. Studies demonstrate that early surgical decompression significantly improves neurological recovery rates, whereas delayed surgery is associated with persistent deficits in up to 40-60% of affected patients [39,43,44]. Persistent spinal instability and neurologic deterioration typically mandate urgent surgical management to prevent worsening disability and to optimize functional outcomes. Careful assessment based on clinical findings and advanced imaging, particularly MRI, which delineates spinal deformity, abscess formation, and vertebral collapse, is essential in guiding surgical decision-making [45].

Discussion

Although non-operative treatment proves effective in approximately 90% of uncomplicated cases, surgical intervention becomes necessary in patients who do not respond to conservative management or present with neurological deficits [1]. In recent years, the percutaneous endoscopic approach has gained traction within the evolving field of spine surgery. This minimally invasive technique has been increasingly utilized for both degenerative spine conditions and infectious spondylitis, particularly in high-risk populations such as the elderly, critically ill, or immunocompromised individuals. Techniques for performing minimally invasive debridement in infectious spondylitis vary across studies. Viezens et al. described a combination of percutaneous debridement and instrumentation, supplemented with a posterior midline approach to perform laminotomy or laminectomy with abscess drainage and disc excision [46]. An anterior approach was then employed using thoracoscopy for levels above L2 and a minimally invasive lumbotomy (pararectal or lateral transpsoas-XLIF) for L2 to S1 levels [46].

In another study, Lin et al. [9] performed a two-stage procedure involving anterolateral interbody fusion and debridement, followed by percutaneous posterior pedicle screw fixation under fluoroscopic guidance. Their approach included Jamshidi needle placement, dilation, cannulation, and rod guidance [9]. Lee et al. utilized a slightly larger 2-3 inch incision for the anterolateral thoracic approach and retroperitoneal access for lumbar cases, followed by chest tube or Hemovac drain placement [47]. Despite the promising outcomes of minimally invasive percutaneous techniques, such as similar fusion rates and favorable early recovery, their lower culture positivity rate (58-90%) compared to open surgery is a concern. This may be attributed to the latter's advantage in directly accessing infected tissue for debridement and microbiological sampling [47]. Nonetheless, Mao et al. reported that PED effectively removes infected material, improves local perfusion, facilitates antibiotic delivery, and results in favorable clinical outcomes [48].

Yang et al. found percutaneous endoscopic spinal procedures satisfactory, though prolonged pain and pre-existing anterior vertebral body damage contributed to extended immobilization and potential complications [17]. Comprehensive debridement and antibiotic therapy are critical for infection control and halting osseous destruction, while instrumentation can facilitate earlier mobilization and mitigate kyphotic progression [17]. A technical comparison by Ahn [49] between unilateral and bilateral PED in 20 patients with lumbar spinal tuberculosis showed that the unilateral method offered shorter operative times and comparable outcomes in terms of inflammatory markers, pain (VAS), functional status (Oswestry Disability Index, ODI), and complication rates.

The findings of this review suggest that MIS significantly reduces operative time compared to open surgery, likely due to quicker paraspinal muscle preparation and wound closure. However, MIS requires longer fluoroscopy time, reflecting the complexity of anatomical visualization and instrumentation, which also increases radiation exposure for surgical staff. These challenges are influenced by the surgeon's learning curve and familiarity with MIS techniques [17,47-49].

MIS also demonstrated a lower complication rate relative to open surgery. Documented MIS-related issues include transient paresthesia, localized infections, kyphosis, and rare reoperations [20,21,23]. Conversely, traditional open procedures are linked to more severe complications such as paraspinal muscle denervation, pleural effusion, and diaphragmatic and vascular injuries, with reported vascular complication rates up to 15% and associated mortality of 1% [20,21]. While reports of PED-specific complications remain scarce, George et al. noted the approach's efficacy and diagnostic utility with no surgery-related complications in both simple and complex infectious spondylodiscitis [1]. However, larger studies are needed to validate these outcomes.

To our knowledge, this is the first comprehensive review to directly compare MIS and OS in infectious spondylitis. While Mao et al. [48] conducted a similar review on PED's efficacy, their study used a single-arm design without direct comparisons. This study has limitations: (1) all included studies are Level III evidence; (2) inclusion of cases with and without instrumentation and staged procedures, which may introduce bias, although baseline characteristics between groups were statistically comparable; and (3) variability in reported MIS incision lengths across studies. Nevertheless, all MIS techniques examined were less invasive than open surgery. The strengths of this study include (1) being the first objective review comparing MIS and open surgery for infectious spondylitis; (2) low heterogeneity (<50%) across eight forest plots, suggesting good representation; and (3) comprehensive outcome assessment across multiple therapeutic dimensions. We hope this review informs future research with larger cohorts and aids in clinical decision-making for the surgical management of infectious spondylitis [48,50].

## Conclusions

In conclusion, MIS of the spine represents a promising alternative treatment for patients with spondylodiscitis, particularly those experiencing neurologic deficits. This comprehensive review of the current literature indicates that MIS techniques consistently yield favorable outcomes in terms of reduced length of hospital stay and lower perioperative morbidity when compared to traditional open surgical approaches. This efficiency results from the inherent advantages of MIS, such as minimized soft tissue trauma, decreased blood loss, and expedited recovery times. Furthermore, in instances where neurologic deficits are present, the ability to achieve adequate neural decompression through a mini-open incision, while mitigating surgical risks, can significantly enhance patient outcomes.

## References

[1] George AJ, Santhanagopal S, Mohan MM, Lal JV, Basappa M, Thomas JC, Jeevo J (2024). Spondylodiscitis: a diagnostic and management dilemma. Cureus.

[2] Verdú-López F, Vanaclocha-Vanaclocha V, Gozalbes-Esterelles L, Sánchez-Pardo M (2014). Minimally invasive spine surgery in spinal infections. J Neurosurg Sci.

[3] Patel PD, Canseco JA, Houlihan N, Gabay A, Grasso G, Vaccaro AR (2020). Overview of minimally invasive spine surgery. World Neurosurg.

[4] Herren C, Jung N, Pishnamaz M, Breuninger M, Siewe J, Sobottke R (2017). Spondylodiscitis: diagnosis and treatment options. Dtsch Arztebl Int.

[5] Zuluaga-García JP, Leon-Aldana S, Herrera D (2025). Spondylodiscitis: a comprehensive review of diagnostic challenges, microbial etiology, and management strategies. SN Compr Clin Med.

[6] Motov S, Stemmer B, Krauss P (2024). Clinical and surgical outcome in patients with cervical spondylodiscitis-a single-center retrospective case series of 24 patients. Front Surg.

[7] Xu J, Zhang L, Bu R, Liu Y, Lewandrowski KU, Zhang X (2021). Minimally invasive debridement and drainage using intraoperative CT-Guide in multilevel spondylodiscitis: a long-term follow-up study. BMC Musculoskelet Disord.

[8] Slowinski J, Lucasti C, Maraschiello M, Kluczynski MA, Kowalski J, Hamill C (2022). Minimally invasive spine surgery as treatment for persistent infectious lumbar spondylodiscitis: a systematic review and meta-analysis. J Spine Surg.

[9] Lin TY, Tsai TT, Lu ML (2014). Comparison of two-stage open versus percutaneous pedicle screw fixation in treating pyogenic spondylodiscitis. BMC Musculoskelet Disord.

[10] Bae JW, Lee SS, Yang JS, Seo EM (2023). Efficacy of minimally invasive oblique lumbar interbody fusion using polyetheretherketone cages for lumbar pyogenic spondylodiscitis treatment. J Pers Med.

[11] Turel MK, Kerolus M, Deutsch H (2017). The role of minimally invasive spine surgery in the management of pyogenic spinal discitis. J Craniovertebr Junction Spine.

[12] Ishihara S, Funao H, Isogai N, Ishihara M, Saito T, Ishii K (2022). Minimally invasive spine stabilization for pyogenic spondylodiscitis: a 23-case series and review of literature. Medicina (Kaunas).

[13] Mooney J, Michalopoulos GD, Alvi MA (2022). Minimally invasive versus open lumbar spinal fusion: a matched study investigating patient-reported and surgical outcomes. J Neurosurg Spine.

[14] Tsai TT, Yang SC, Niu CC, Lai PL, Lee MH, Chen LH, Chen WJ (2017). Early surgery with antibiotics treatment had better clinical outcomes than antibiotics treatment alone in patients with pyogenic spondylodiscitis: a retrospective cohort study. BMC Musculoskelet Disord.

[15] Hinojosa-Gonzalez DE, Roblesgil-Medrano A, Villarreal-Espinosa JB (2022). Minimally invasive versus open surgery for spinal metastasis: a systematic review and meta-analysis. Asian Spine J.

[16] Yuan S, Ma F, Wang Y, Gong P (2019). Minimally invasive spine surgery in the treatment of pyogenic spondylodiscitis: an initial retrospective series study. Wideochir Inne Tech Maloinwazyjne.

[17] Yang SC, Fu TS, Chen HS, Kao YH, Yu SW, Tu YK (2014). Minimally invasive endoscopic treatment for lumbar infectious spondylitis: a retrospective study in a tertiary referral center. BMC Musculoskelet Disord.

[18] Wang X, Zhou S, Bian Z, Li M, Jiang W, Hou C, Zhu L (2018). Unilateral percutaneous endoscopic debridement and drainage for lumbar infectious spondylitis. J Orthop Surg Res.

[19] Dakwar E, Cardona RF, Smith DA, Uribe JS (2010). Early outcomes and safety of the minimally invasive, lateral retroperitoneal transpsoas approach for adult degenerative scoliosis. Neurosurg Focus.

[20] Wang SF, Tsai TT, Li YD (2022). Percutaneous endoscopic interbody debridement and fusion (PEIDF) decreases risk of sepsis and mortality in treating infectious spondylodiscitis for patients with poor physical status, a retrospective cohort study. Biomedicines.

[21] Lai PJ, Wang SF, Tsai TT, Li YD, Chiu PY, Hsieh MK, Kao FC (2021). Percutaneous endoscopic interbody debridement and fusion for pyogenic lumbar spondylodiskitis: surgical technique and the comparison with percutaneous endoscopic drainage and debridement. Neurospine.

[22] Abe K, Orita S, Mannoji C (2017). Perioperative complications in 155 patients who underwent oblique lateral interbody fusion surgery: perspectives and indications from a retrospective, multicenter survey. Spine (Phila Pa 1976).

[23] Tschugg A, Hartmann S, Lener S, Rietzler A, Sabrina N, Thomé C (2017). Minimally invasive spine surgery in lumbar spondylodiscitis: a retrospective single-center analysis of 67 cases. Eur Spine J.

[24] Olsen MA, Mayfield J, Lauryssen C, Polish LB, Jones M, Vest J, Fraser VJ (2003). Risk factors for surgical site infection in spinal surgery. J Neurosurg.

[25] Schömig F, Putzier M (2020). Clinical presentation and diagnosis of delayed postoperative spinal implant infection. J Spine Surg.

[26] Schimmel JJ, Horsting PP, de Kleuver M, Wonders G, van Limbeek J (2010). Risk factors for deep surgical site infections after spinal fusion. Eur Spine J.

[27] Pull ter Gunne AF, Cohen DB (2009). Incidence, prevalence, and analysis of risk factors for surgical site infection following adult spinal surgery. Spine (Phila Pa 1976).

[28] Kasliwal MK, Tan LA, Traynelis VC (2013). Infection with spinal instrumentation: review of pathogenesis, diagnosis, prevention, and management. Surg Neurol Int.

[29] Kim SJ, Lee SH, Chung HW, Lee MH, Shin MJ, Park SW (2017). Magnetic resonance imaging patterns of post-operative spinal infection: relationship between the clinical onset of infection and the infection site. J Korean Neurosurg Soc.

[30] Mok JM, Pekmezci M, Piper SL (2008). Use of C-reactive protein after spinal surgery: comparison with erythrocyte sedimentation rate as predictor of early postoperative infectious complications. Spine (Phila Pa 1976).

[31] Thavarajasingam SG, Vemulapalli KV, Vishnu K S (2023). Conservative versus early surgical treatment in the management of pyogenic spondylodiscitis: a systematic review and meta-analysis. Sci Rep.

[32] Singh DK, Singh N, Das PK, Malviya D (2018). Management of postoperative discitis: a review of 31 patients. Asian J Neurosurg.

[33] Blecher R, Frieler S, Qutteineh B (2023). Who needs surgical stabilization for pyogenic spondylodiscitis? Retrospective analysis of non-surgically treated patients. Global Spine J.

[34] Taylor DG, Buchholz AL, Sure DR (2018). Presentation and outcomes after medical and surgical treatment versus medical treatment alone of spontaneous infectious spondylodiscitis: a systematic literature review and meta-analysis. Global Spine J.

[35] Pola E, Autore G, Formica VM, Pambianco V, Colangelo D, Cauda R, Fantoni M (2017). New classification for the treatment of pyogenic spondylodiscitis: validation study on a population of 250 patients with a follow-up of 2 years. Eur Spine J.

[36] Poutoglidou F, Metaxiotis D, Saloupis P, Mpeletsiotis A (2021). Operative treatment of adult pyogenic spondylodiscitis: a retrospective study of 32 cases. Cureus.

[37] Kramer A, Thavarajasingam SG, Neuhoff J, Davies BM, Demetriades AK, Shiban E, Ringel F (2024). Variation of practice in the treatment of pyogenic spondylodiscitis: a European Association of Neurosurgical Societies Spine Section study. J Neurosurg Spine.

[38] Patel AR, Alton TB, Bransford RJ, Lee MJ, Bellabarba CB, Chapman JR (2014). Spinal epidural abscesses: risk factors, medical versus surgical management, a retrospective review of 128 cases. Spine J.

[39] Nabizadeh N, Crawford CH 3rd, Glassman SD, Dimar II JR, Carreon LY (2022). Severity and outcome of neurologic deficits in patients with pyogenic spondylodiscitis: a systematic review. Orthop Clin North Am.

[40] Bettini N, Girardo M, Dema E, Cervellati S (2009). Evaluation of conservative treatment of non specific spondylodiscitis. Eur Spine J.

[41] Lener S, Hartmann S, Barbagallo GM, Certo F, Thomé C, Tschugg A (2018). Management of spinal infection: a review of the literature. Acta Neurochir (Wien).

[42] Lima D, Lopes N, Pereira AL, Rodrigues D, Amaral-Silva M, Marques E (2024). Diagnosis and treatment of spondylodiscitis: insights from a five-year single-center study. Cureus.

[43] Tan Y, Mohamed Ramlee FA, Harun MH, Jaapar MS, Tan CN (2024). Clinical and radiological outcomes of extreme lateral interbody fusion (XLIF) in the treatment of lumbar spondylodiscitis: a multi-center study. Cureus.

[44] Ferri I, Ristori G, Lisi C, Galli L, Chiappini E (2020). Characteristics, management and outcomes of spondylodiscitis in children: a systematic review. Antibiotics (Basel).

[45] Maamari J, Grach SL, Passerini M (2023). The use of MRI, PET/CT, and nuclear scintigraphy in the imaging of pyogenic native vertebral osteomyelitis: a systematic review and meta-analysis. Spine J.

[46] Viezens L, Schaefer C, Helmers R, Vettorazzi E, Schroeder M, Hansen-Algenstaedt N (2017). Spontaneous pyogenic spondylodiscitis in the thoracic or lumbar spine: a retrospective cohort study comparing the safety and efficacy of minimally invasive and open surgery over a nine-year period. World Neurosurg.

[47] Lee CY, Huang TJ, Li YY, Cheng CC, Wu MH (2014). Comparison of minimal access and traditional anterior spinal surgery in managing infectious spondylitis: a minimum 2-year follow-up. Spine J.

[48] Mao Y, Li Y, Cui X (2019). Percutaneous endoscopic debridement and drainage for spinal infection: systemic review and meta-analysis. Pain Physician.

[49] Ahn Y (2012). Transforaminal percutaneous endoscopic lumbar discectomy: technical tips to prevent complications. Expert Rev Med Devices.

[50] Peters DR, Owen T, Hani U (2024). Open versus percutaneous stabilization of thoracolumbar fractures: a large retrospective analysis of safety and reoperation rates. Cureus.

